# Mechanisms of Tolerance and Resistance to Chlorhexidine in Clinical Strains of *Klebsiella pneumoniae* Producers of Carbapenemase: Role of New Type II Toxin-Antitoxin System, PemIK

**DOI:** 10.3390/toxins12090566

**Published:** 2020-09-02

**Authors:** Ines Bleriot, Lucia Blasco, Mercedes Delgado-Valverde, Ana Gual-de-Torrella, Anton Ambroa, Laura Fernandez-Garcia, Maria Lopez, Jesus Oteo-Iglesias, Thomas K. Wood, Alvaro Pascual, German Bou, Felipe Fernandez-Cuenca, Maria Tomas

**Affiliations:** 1 Microbiology Department-Research Institute Biomedical A Coruña (INIBIC), Hospital A Coruña (CHUAC), University of A Coruña (UDC), 15006 A Coruña, Spain; bleriot.ines@gmail.com (I.B.); luciablasco@gmail.com (L.B.); anton17@mundo-r.com (A.A.); laugemis@gmail.com (L.F.-G.); maria.lopez.diaz@sergas.es (M.L.); German.Bou.Arevalo@sergas.es (G.B.); 2 Study Group on Mechanisms of Action and Resistance to Antimicrobials (GEMARA) the Behalf of the Spanish Society of Infectious Diseases and Clinical Microbiology (SEIMC), 28003 Madrid, Spain; jesus.oteo@isciii.es(J.-O.I.); apascual@us.es (A.P.); felipefc@us.es (F.F.-C.); 3 Clinical Unit for Infectious Diseases, Department of Microbiology and Medicine, Microbiology and Preventive Medicine, Hospital Universitario Virgen Macarena, University of Seville, Biomedicine Insititute of Seville (IBIS), 41009 Seville, Spain; mercdss@gmail.com (M.D.-V.); gualdetorrella.ana@gmail.com (A.G.-d.-T.); 4 Spanish Network for Research in Infectious Diseases (REIPI), 41071 Seville, Spain; 5 Reference and Research Laboratory for Antibiotic Resistance and Health Care Infections, National Centre for Microbiology, Institute of Health Carlos III, 28222 Majadahonda, Spain; 6 Department of Chemical Engineering, Pennsylvania State University, University Park, PA 16801, USA; tuw14@psu.edu

**Keywords:** tolerance, persistence, cross-resistance, toxin-antitoxin system, PemI/PemK, *Klebsiella pneumoniae*

## Abstract

Although the failure of antibiotic treatment is normally attributed to resistance, tolerance and persistence display a significant role in the lack of response to antibiotics. Due to the fact that several nosocomial pathogens show a high level of tolerance and/or resistance to chlorhexidine, in this study we analyzed the molecular mechanisms associated with chlorhexidine adaptation in two clinical strains of *Klebsiella pneumoniae* by phenotypic and transcriptomic studies. These two strains belong to ST258-KPC3 (high-risk clone carrying β-lactamase KPC3) and ST846-OXA48 (low-risk clone carrying β-lactamase OXA48). Our results showed that the *K. pneumoniae* ST258-KPC3CA and ST846-OXA48CA strains exhibited a different behavior under chlorhexidine (CHLX) pressure, adapting to this biocide through resistance and tolerance mechanisms, respectively. Furthermore, the appearance of cross-resistance to colistin was observed in the ST846-OXA48CA strain (tolerant to CHLX), using the broth microdilution method. Interestingly, this ST846-OXA48CA isolate contained a plasmid that encodes a novel type II toxin/antitoxin (TA) system, PemI/PemK. We characterized this PemI/PemK TA system by cloning both genes into the IPTG-inducible pCA24N plasmid, and found their role in persistence and biofilm formation. Accordingly, the ST846-OXA48CA strain showed a persistence biphasic curve in the presence of a chlorhexidine-imipenem combination, and these results were confirmed by the enzymatic assay (WST-1).

## 1. Introduction

The increase in antimicrobial resistance due to the emergence of multi-drug resistant (MDR) pathogens is one of the world’s greatest public health challenges, as it can lead to an era without effective antibiotics [1]. Recently, the World Health Organization (WHO) published a list of “priority pathogens”, which includes those microorganisms considered a serious threat to human health. Some members of this list are carbapenem-resistant pathogens and are known under the acronym of ESKAPE, including among other species, *Klebsiella pneumoniae* [1,2,3]. *K. pneumoniae* is a Gram-negative, opportunistic bacteria pathogen associated with a wide range of diseases such as urinary tract infections, pneumoniae, septicemia, wounds, and soft tissue infections [4]. Carbapenem resistance is increasing rapidly worldwide, particularly among *K. pneumoniae*. The main carbapenem-resistance mechanism is acquisition of plasmid-encoded carbapenemases, which may belong to the molecular class A (i.e., KPC- type), B (i.e., imipenem (IMP)-type, VIM-type, NDM-type) and D (i.e., OXA-48-type). The high-risk clones of *K. pneumoniae*, in contrast to low-risk clones, have an extraordinary ability to persist and spread in the nosocomial environment, disseminating these carbapenemases and therefore being involved in nosocomial outbreaks [4].

Nevertheless, much less attention has been paid to the presence or occurrence of resistance to antiseptics and biocides, such as chlorhexidine (CHLX) [5], widely used in hospital settings. CHLX is a symmetric bis-biguanide molecule comprising two chloroguanide chains that are connected by a central hexamethylene chain, and carry two positive charges at physiological pH. CHLX is sparingly soluble in water, and thereby normally formulated with either acetate or gluconate to form water-soluble salts [6]. The antimicrobial effect of this compound is based on damaging the bacterial membrane, leading to the subsequent leakage of cytoplasmatic material. Therefore, mechanisms conferring resistance toward CHLX include multidrug efflux pumps and cell membrane changes [5]. Moreover, CHLX adaptation has been associated with the emergence of stable resistance to the last-resort antibiotic colistin (polymyxin E) [7,8,9].

In general, the failure of antibiotic treatments has been associated with resistance mechanisms. However, it has recently been noted that other mechanisms such as tolerance and persistence were also involved [10]. The recovery of persistent cells is one of the main causes of prolonged and recurrent infections, that can lead to the complete failure of antibiotic treatments [11]. In this context, it is important to distinguish between resistant, tolerant, and persistent bacteria [12]. The term resistance is generally used to describe the inherited ability of a bacterial population to grow in the presence of high concentrations of antibiotics, regardless of the duration of treatment [12], due to active defense mechanisms associated with mutations [10]. Whereas, the term tolerance is used to describe the ability, inherited or not, of a bacterial population to survive the transient exposure of high concentrations of antibiotics without causing changes in minimum inhibitory concentrations (MICs), due to the deceleration of essential biological processes [10,11,12]. It is important to emphasize that despite the slow-growth rate, tolerant bacteria keep a metabolically active state. In contrast to resistance and tolerance, persistence is characterized by the ability, not inherited, of a bacterial subpopulation (around 0.001–1%) [10] to resist antibiotics by growth arrest due to the inactivation of their metabolism and their non-replicative state, thus it is due to their dormant state. Persistent bacteria exhibit transient levels of tolerance to antibiotics that do not affect their MICs, so once the drug pressure is removed and their metabolism is reactivated, they can rapidly re-grow. Nowadays, it is known that multiple molecular mechanisms are involved in the formation of persistent bacteria such as the stringent response molecule (p)ppGpp, stress response, SOS response, quorum sensing, toxin-antitoxin (TA) systems, efflux pumps, the ROS response and energy metabolism, among others [11]. 

The involvement of TA systems in cell physiology, specifically in: (i) biofilm formation by regulating fimbriae [13,14], (ii) bacterial persistence, by generating slowly-growing cells tolerant to antibiotics and environmental changes [15,16,17,18,19], (iii) plasmid maintenance [20,21], (iv) general stress response [22], and (v) phage inhibition [23,24,25] is becoming clearer [18]. A TA system is a module of two genes encoding a stable toxin and an unstable antitoxin. Under normal growth conditions the antitoxin inhibits the toxin, but under stress conditions the antitoxin is degraded, leaving the toxin free to inhibit the basic cellular processes like DNA replication or protein synthesis, and also promoting plasmid maintenance, slow growth and latency [18]. These systems are widely distributed and found in the bacterial chromosome, plasmids, and bacteriophages [2]. 

In this context, this study provides a better comprehension of the molecular mechanisms associated with chlorhexidine adaptation (CA) in two clinical strains of *K. pneumoniae*, both of which produce carbapenemase: ST258-KPC3 (high-risk clone carrying β-lactamase KPC3) and ST846-OXA48 (low-risk clone carrying β-lactamase OXA48), from a phenotypic and transcriptomic point of view. It should be noted that international high-risk clones of *K. pneumoniae* are among the most common nosocomial pathogens. The success of these clones is due to their facility to spread their plasmids, which carry a considerable variety of antimicrobial resistance genes [26,27]. Thus, the study of this type of clones is of great clinical relevance. Moreover, this study aims to characterize a new toxin-antitoxin system (PemIK) located in a plasmid inside the ST846-OXA48CA strain, and to examine the possible role of this system in persistence and in biofilm formation.

## 2. Results

### 2.1. Results Subsection

#### 2.1.1. Time-Killing Curve in the Presence of CHLX (10 × MIC)

**Figure 1:**
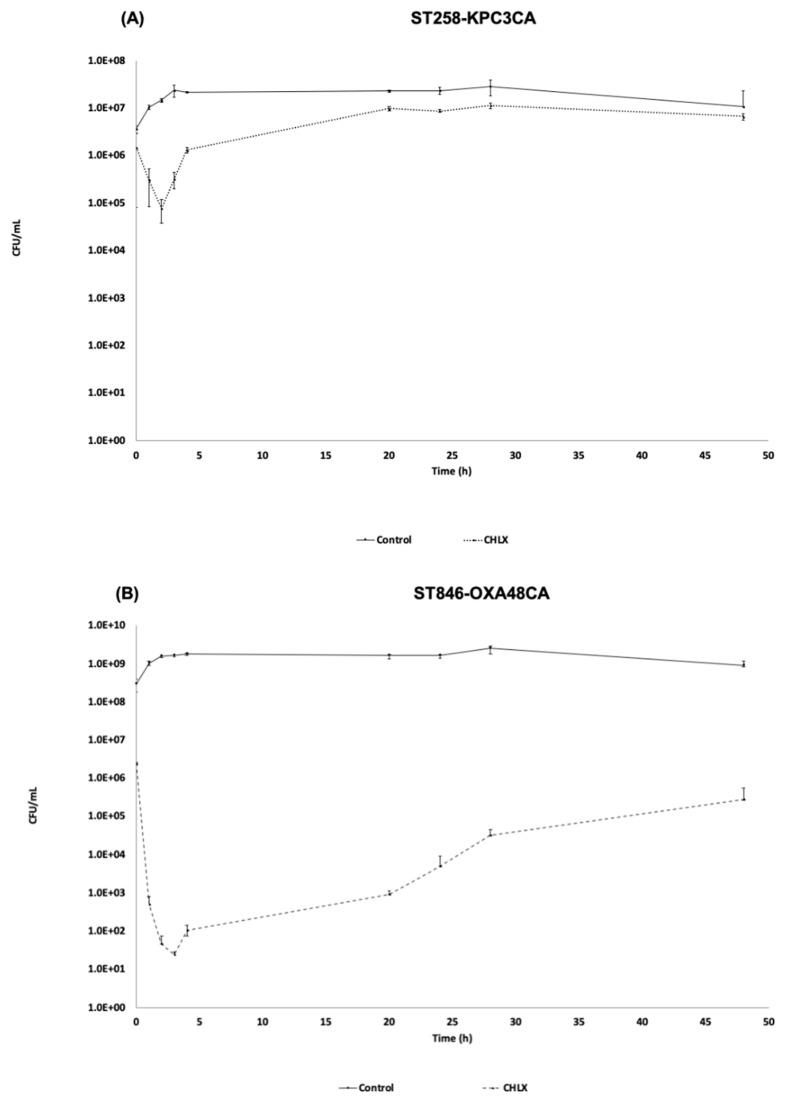
Time-killing curve in the presence of chlorhexidine (CHLX) (10 × minimum inhibitory concentration (MIC)) in *K. pneumoniae* chlorhexidine adaptation (CA) strains ST258-KPC3CA (**A**) and ST846-OXA48CA (**B**). The same strains without being exposed to biocide pressure are used as controls. The errors bar represents the standard deviation of the three replicates experiment.

The time-killing curves of the strains ST258-KPC3CA and ST846-OXA48CA in the presence of CHLX (10 × MIC) showed two different growth patterns (Figure 1). The strain ST258-KPC3CA showed a slight reduction in its bacterial population, occurring in the first two hours of CHLX exposure, decreasing from 6 LogCFU/mL (1.47 × 10^6^ CFU/mL) to 4 LogCFU/mL (7.75 × 10^4^ CFU/mL) at 2 h (Figure 1A). This slight reduction in the bacterial population occurs during the activation of defense mechanisms, such as efflux pumps, which can reduce the effective concentration of the drug in the cell. In contrast, the ST846-OXA48CA strain, dramatically reduced its bacterial population during the first four hours of CHLX exposure, decreasing from of a bacterial population of 6 LogCFU/mL (2.43 × 10^6^ CFU/mL) to 2 LogCFU/mL (1.05 × 10^2^ CFU/mL) at 4 h (Figure 1B). After this period, both bacterial strains grew again reaching respectively a bacterial population of 6 LogCFU/mL (6.63 × 10^6^ CFU/mL) and 5 LogCFU/mL (2.75 × 10^5^ CFU/mL) at 48 h. The curves have the characteristics of a resistant strain in the first case (ST258-KPC3CA) and a tolerant strain in the second one (ST846-OXA48CA), according to the definition of each mechanisms. Resistance is the ability of bacterial population to grow at a similar rate in the presence of an environmental stress, while the tolerance is the ability of a bacterial population to withstand the stress.

#### 2.1.2. Transcriptomic Study

All the transcriptomic results are deposited in the NCBI database as a GenBank BioProject (Code number: PRJNA609262) and GEO series (Code number: GSE147316). The transcriptomic profile from the ST258-KPC3CA isolate indicated a probable CHLX resistant profile. Indeed, this strain has a higher number of overexpressed genes (Log2fold change > 1.5), especially for transporters and efflux pumps such as the methyl viologen resistance gene *smvA* (Log2fold change: 3.635), which is involved in the cationic biocide resistance. However, strain ST846-OXA48CA showed what it was as a CHLX tolerant profile, with repressed genes (Log2FoldChange < 1.5) for efflux pumps, TA systems, SOS response, and ppGpp mechanisms (Table 1). This strain also showed high levels of expression of genes, *pmrD*, and *pmrK* (Log2fold change 2.360 and 1.570, respectively), characteristics of the colistin resistance. Therefore, these transcriptomic results corroborated the results obtained by the time-killing curves, showing activation of molecular mechanisms of resistance and tolerance molecular mechanisms in response to CHLX in ST258-KPC3CA and ST846-OXA48CA strains, respectively.

#### 2.1.3. Antimicrobial Susceptibly Testing

The antimicrobial susceptibility test was done for wild-type ST258-KPC3 and ST846-OXA48 wild-type and the two CA strains. According to adaptation to CHLX, an increase in MICs of CHLX was observed in both CA strains. However, no differences in the minimum inhibitory concentration (MIC) values were observed for the other antibiotics tested, except for the colistin, in that in the ST846-OXA48CA strain showed an increase in the MIC value of 32-fold, that corresponds to a resistance value (Table 2).

#### 2.1.4. Characterization of the New TA System, PemI/PemK, Present in a Plasmid in the Strain ST846-OXA48CA

**Figure 2:**
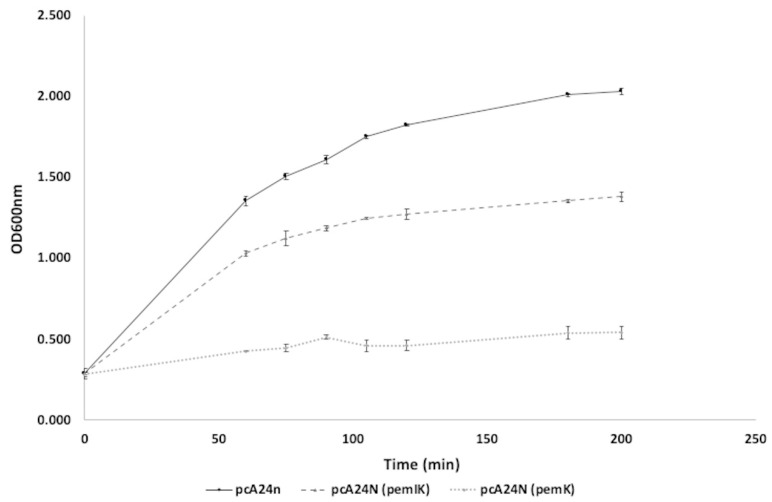
Growth curves of ST846-OXA48CA CP containing pCA24N plasmids with *pemIK* (dark grey, dashed line) and *pemK* (light grey, dotted line) in the presence of 1 mM IPTG. The strain ST846-OXA48CA CP is used as a control as it carries the empty plasmid pCA24N (black). The errors bar represents the standard deviation of the three experimental replicates.

A PemI/PemK TA system, whose closest relative is the type II TA toxin-antitoxin system PemK/MazF family toxin belonging to Enterobacteriacea (Query: 78%; Identity: 99.35%; Code number: WP_077688581.1) and which have not been previously described in *K. pneumoniae*, it was identified by transcriptomic analysis (Table 1) as encoded by a plasmid of *K. pneumoniae* ST846-OXA48CA (Figure S1). This plasmid harbors several genes, such as *repA*, *dsbC*, *trbA*, *trbC*, *lusR*, CPBP metalloprotease, *umuD*, *umuC*, restriction endonuclease, *IS1*, *ssb*, *mobC, nikA*, *dotD*/TraH family lipoprotein, secretion systems type IV, *traO*, *traP*, *traQ*, *traW*, *traX*, *dotA*/*traY*, and *repC*, in addition to another TA system, the RelE/RelB TA system. This PemI/PemK TA system is composed of a 258 bp antitoxin gene (*pemI*) and a 333 bp toxin gene (*pemK*). To confirm that this system is a TA system, *pemI*/*pemK* and *pemK* genes alone were cloned into the overexpression vector pCA24N, widely used in the literature to overexpress TA systems [28,29], and transformed into the cured plasmid strain ST846-OXA48CA CP (i.e., lacking plasmids and therefore the plasmid that encodes the PemI/PemK TA system). The toxicity of this TA system was tested by growth curves overexpressing both *pemI*/*pemK* and *pemK* (Figure 2). Overexpression of *pemK* in ST846-OXA48CA CP/pCA24N (*pemK*) inhibited bacterial growth, while overexpression of the *pemI*/*pemK* system (ST846-OXA48CA CP/pCA24N (*pemIK*)) in the strain led to normal bacterial growth, which was slightly impaired compared to the empty plasmid. Therefore, the plasmid-based PemI/PemK TA system found in *K. pneumoniae* ST846-OXA48CA is functional.

#### 2.1.5. Biofilm Formation Assay

**Figure 3:**
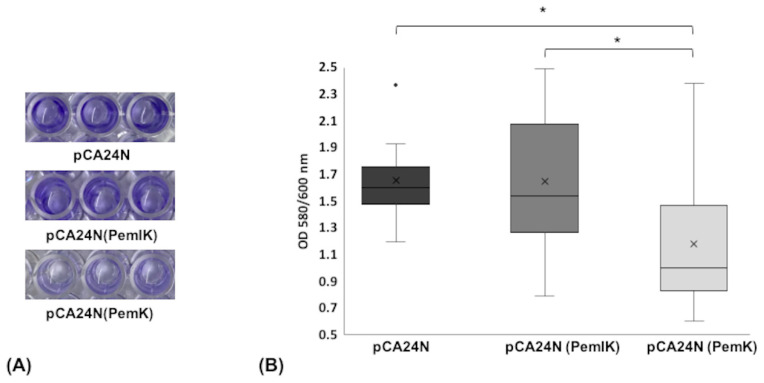
Biofilm formation assay. (**A**) Biofilm strained with 10% crystal violet was dissolved in 30% acetic acid. (**B**) Box and whisker plot of the optical density of biofilm produced by the strains ST846-OXA48CA CP/pCA24N, ST846-OXA48CA CP/pCA24N (PemIK), and ST846-OXA48CA CP/pCA24N (PemK). The biofilm formation was expressed as the ratio between OD580/600 nm, in order to normalize the data. Boxes indicate the lower and upper quartile. Horizontal lines in each box represents the median value of biofilm formation. The mean biofilm formation for each strain is indicated by a +. Vertical lines extending from each box represent the minimum and maximum biofilm formation. *, *p*-value < 0.05. All experiments were performed in triplicates.

Since TA systems have been associated with the arrest of bacterial growth and the formation of biofilms, we studied the effect of the PemI/PemK TA system and the PemK toxin on biofilm formation (Figure 3). Production of PemK toxin resulted in a significant decrease in biofilm formation compared to the control (ST846-OXA48CA CP/pCA24N) (*p*-value < 0.001). Moreover, production of PemI/PemK restored a similar phenotype as the control, lacking a significant difference in biofilm formation (*p*-value < 0.05). Therefore, the PemI/PemK TA system influences *K. pneumoniae* biofilm formation.

#### 2.1.6. Time-Killing Curve in the Presence of Imipenem or in Combination with Chlorhexidine for ST846-OXA48CA and ST846-OXA48CA CP

**Figure 4:**
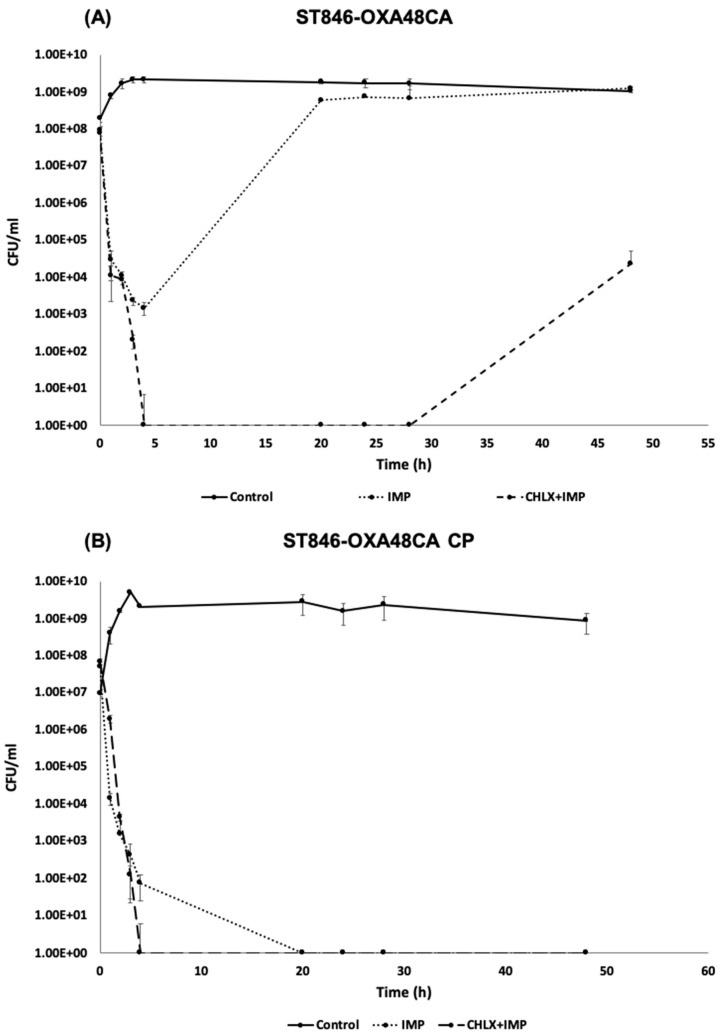
Time-killing curve in the presence of IMP (50 × MIC) and in presence of the combination of IMP (50 × MIC) and CHLX (10 × MIC) for the strains of *K. pneumoniae* ST846-OXA48CA (**A**) and ST846-OXA48CA CP (**B**). The controls are the strains without exposure to any stress (IMP, IMP + CHLX). The error bars represent the standard deviation of the three replicates of the experiment.

The time-killing curves of *K. pneumoniae* ST846-OXA48CA tolerant to CHLX (Figure 4A) were performed in the presence of imipenem (IMP) (50 × MIC) alone or in combination with CHLX (10 × MIC). A drastic reduction in the number of CFU was observed during the first four hours in the presence of IMP (50 × MIC), decreasing from 7 LogCFU/mL (9.35 × 10^7^ CFU/mL) to 3 LogCFU/mL (1.50 × 10^3^ CFU/mL). However, bacterial regrowth occurred after four hours, reaching similar levels of CFU/mL as the control (8 LogCFU/mL (6.83 × 10^8^ CFU/mL) vs. 9 LogCFU/mL (1.73 × 10^9^ CFU/mL)) at 28 h and exceeding it at 48 h (9 LogCFU/mL (1.25 × 10^9^ CFU/mL) vs. 9 LogCFU/mL (1.07 × 10^9^ CFU/mL)). In the case of the combination of IMP and CHLX, a greater CFU reduction than IMP alone was observed, with no CFU detected after 4 h. Nevertheless, a regrowth of the bacterial population was observed after 28 h. Thus, ST846-OXA48CA in presence of the combination of IMP and CHLX showed a characteristic behavior of the persistent subpopulation.

In the case of the ST846-OXA48CA CP strain (Figure 4B), in which the plasmid was removed by means of a curing agent, 3% sodium dodecyl sulfate (SDS) (10% *w*/*v* pH = 7.4), a drastic reduction in the number of CFU, was observed both with IMP alone as for in the combination of IMP and CHLX. In fact, in the case of IMP alone no CFUs were recovered at 20 h while, in the case of the combination, no CFUs were recovered at 4 h (Figure 4). Finally, the culture was considered dead as no regrowth was observed throughout the rest of the assay. Thus, the ST846-OXA48CA CP strain, unlike the ST846-OXA48CA strain, showed more sensitive behavior curve pattern in the presence of IMP alone and in the presence of the combination of drug. These results may suggest that the absence of the plasmid containing both the β-lactamase OXA48 and the TA system PemI/PemK could be a factor responsible for the absence of regrowth. Moreover, the lack of the TA system PemI/PemK could be implicated in the non-appearance of a persistent subpopulation in presence of the combination of IMP and CHLX, contrary to what happens in the strain ST846-OXA48 CA.

#### 2.1.7. Enzymatic Analysis Using the Cell Proliferation Reagent WST-1 

**Figure 5:**
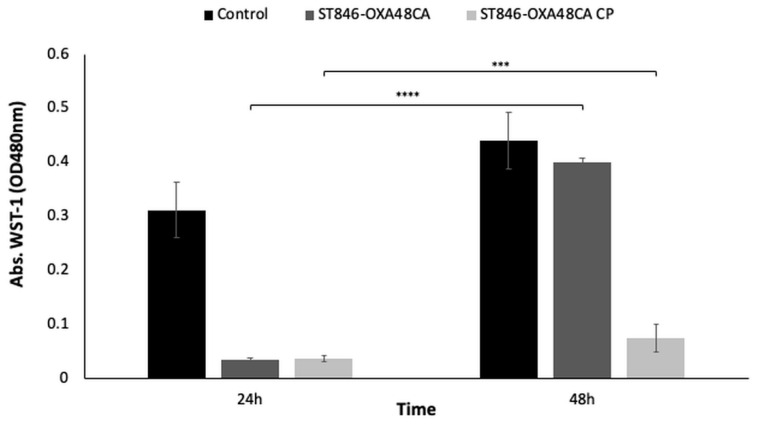
Enzymatic activity by the colorimetric assay (WST-1 based) of the strain *K. pneumoniae* ST846-OXA48CA and ST846-OXA48CA CP in the presence of the combination of IMP (50 × MIC) and CHLX (10 × MIC). The growth control is ST846-OXA48CA strain without antibiotic pressure. ***, *p*-value < 0.001 and ****, *p*-value < 0.0001. The errors bars represent the standard deviation of the three experiment replicates.

The results of the time-killing curves in the presence of the IMP and CHLX combination was confirmed by enzymatic analysis using the cell proliferation reagent WST-1 (Figure 5), which measures the omnipresent reducing agents NADH and NADPH as biochemical markers to evaluate the metabolic activity of the cell [30]. Indeed, ST846-OXA48CA lacks metabolic activity/cell proliferation at 24 h (OD_480 nm_ < 0.01), whereas it presents a significant increase at 48 h (OD_480 nm_ > 0.4; *p*-value < 0.0001), confirming regrowth in the bacterial culture. In contrast, the ST846-OXA48CA CP strain, despite showing significant differences (*p*-value < 0.002) between 24 and 48 h in terms of metabolic activity/cell proliferation, is considered as a dead culture since its OD_480 nm_ is less than 0.1.

## 3. Discussion

Due to the emergence of MDR pathogens over the past few decades, public health officials faces new challenges, such as the alarming increase in antimicrobial resistance, as well as the emerging link between resistance strategies used by bacteria against antibiotics and biocides [18]. This last problem is even more worrisome due to the routinely and uncontrolled use of antiseptics and biocides in clinical practice [9]. One example of this is CHLX, a bis-biguanide antiseptic of cationic nature that has bactericidal activity through membrane disruption [31]. For these reasons it of great interest to decipher the molecular mechanisms involved in the adaptation to CHLX in clinical strains of *K. pneumoniae*, producers of carbapenemases.

In order to determine at the molecular level the effect of the adaptation to CHLX in a strains of *K. pneumoniae*, we performed a phenotypic study in the presence of CHLX (10 × MIC), which showed that the adaptation to CHLX led to the activation of two different molecular mechanisms in the clinical strains of *K. pneumoniae* ST258-KPC3CA and ST846-OXA48CA. In effect, the ST258-KPC3CA strain presented a growth curve typical of resistant bacteria, where a slight reduction in the bacterial population occurs during the time of activation of defense mechanisms [32] (e.g., efflux pumps, TA systems, quorum network), followed by a regrowth period similar to the control. In contrast, ST846-OXA48CA had a characteristic growth curve of tolerant bacteria, where the strain undergoes a drastic reduction or arrest of growth during the first four hours of exposure to the bactericide [31]. These results were corroborated by the transcriptomic study where the transcriptomic profile of the ST258-KPC3CA strain revealed the overexpression of a larger number of genes compared to ST846-OXA48CA strain, especially those related to the overexpression of efflux pumps, which are generally considered as a basic molecular mechanism associated with resistance [33]. In our study, the efflux pump gene that was overexpressed in ST258-KPC3CA was the methyl viologen resistance gene *smvA*. SmvA is responsible for resistance to CHLX in *K. pneumoniae* strains previously adapted to CHLX, due to the interruption of the *smvR* gene (*smvA* repressor) [9].

The antimicrobial susceptibility test revealed the appearance of cross-resistance to colistin (polymyxin E), in the ST846-OXA48CA strain tolerant to CHLX, increasing its MIC value 32-fold. These results were also corroborated by the transcriptomic studies, in which a high level of expression of the colistin resistance genes, *pmrD* and *pmrK* could be observed. This phenomenon of cross-resistance was also described previously, where five out of six strains of *K. pneumoniae* adapted to CHLX also presented resistance to colistin, increasing their MIC values from 2–4 mg/L to 64 mg/L [9]. This phenotype is due to the common biochemical characteristics of CHLX and colistin: in fact, both are cationic compounds with hydrophobic functions [6]. As for the other antibiotics tested, no change in the MIC value was observed in the strain adapted to CHLX compared to the wild-types. 

In the previous study of Fernández-García et al. (2018), a combination of β-lactam antibiotic IMP with the CHLX biocide caused in some strains of *Acinetobacter baumannii* the formation of a subpopulation of persistent bacteria [12]. This phenomenon could be observed in the ST846-OXA48CA strain in the presence of the same combination. However, we have seen that the combination of this β-lactam antibiotic and the biocide did not lead to the appearance of a persistent subpopulation in ST846-OXA48 CA CP, cured of the plasmid, but rather led to the death of the bacterial culture after 4 h. This suggests that the presence of the TA systems carried by the plasmid, PemI/PemK and RelE/RelB, could be responsible of the emergence of a persistent subpopulation. As it has long been shown that TA systems are involved in the formation of persistent bacterial subpopulations [19,34,35]; indeed, TA systems are genetic elements composed of a toxin, which inhibits bacterial growth by interfering with essential cellular processes, and an antitoxin, which is able to neutralize the effect of the toxin in normal growth conditions [11,18,36]. The *pemI*/*pemK* genetic module present in the plasmid of *K. pneumoniae* ST846-OXA48CA, has never been described in *K. pneumoniae.* This TA system was characterized and overexpression assays confirmed that this module corresponds to a TA system. The overexpression of the *pemK* gene led to the inhibition of bacterial growth; however, overexpression of the *pemIK* module led to normal bacterial growth. The same phenomenon was observed in *Bacillus anthracis* where the overexpression of *pemK* in the pHCMC05 vector was severely toxic to the growth of *B. anthracis* cells [37]. 

In recent decades, many studies have shown that TA systems are associated with the formation of biofilms [22,38]. Biofilms are characterized by a dense multicellular community of microorganisms, constituted after the attachment of bacteria to a biotic or an abiotic surface [39]. In fact, the first TA system linked to biofilm formation was the MqsR/MqsA system of *Escherichia coli* [13]. The toxins have been described as modulators of biofilm formation [13,40]. In this study, we have seen that the overexpression of the PemK toxin contributed to a significant decrease in biofilm formation. The effect that we observed for the toxin is corroborated by the study of García-Contreras et al. (2008), [41], where the *hha*-deletion mutant significantly increased biofilm formation. In addition, the complemented *hha* mutant showed a consistently and dramatically inhibition of biofilm formation. Besides, Ma et al. (2019), described that the disruption of MazF toxin, in *Staphylococcus aureus*, led to an increase in biofilm formation in an *ica*-cluster dependent way, as the disruption of *mazF* produced an increase in the level of expression of *icaA*, *icaB*, and *icaC* genes [42]. Furthermore, in the study of Kim et al. (2009), the authors described that the overexpression of five toxins conduced to a decrease in biofilm formation at 8 h, although at 24 h they observed an increase in biofilm [14].

## 4. Conclusions

This is the first study that describes the different effects of the adaptation to CHLX in two clinical strains of *K. pneumoniae*, producers of carbapenemase, that become resistant (ST258-KPC3CA) and tolerant (ST846-OXA48CA) to CHLX. This adaptation has lead, in the case of ST846-OXA48CA strain, to the development of cross-resistance to colistin, an antibiotic of last resort in hospital infections. 

Furthermore, this study is the first one to describe the relationship between the mechanisms of bacterial persistence and the combination of a β-lactam antibiotic (IMP) and a biocide (CHLX) in the clinical isolate of *K. pneumoniae* ST846-OXA48CA. Finally, a new PemI/PemK TA system was identified in a plasmid of the ST846-OXA48CA strain. Its subsequent characterization demonstrated it participates in the development of persister cells as well as the establishment of biofilms.

## 5. Materials and Methods

### 5.1. Bacterial Strains and CHLX Adaptation

Two clinical strains of *K. pneumoniae*, producers of carbapenemases harbored in plasmids, ST258-KPC3 (high-risk clone) and ST846-OXA48 (low-risk clone) were used in this study. These clinical strains belonging to different sequence types (ST) and are from urine and sputum samples, respectively. The attribution of the ST was carried out in the study of Esteban-Cantos et al. (2017) according to the scheme of the Pasteur Institute (http://bigsdb.web.pasteur.fr/Klebsiella/) [43].

In order to obtain the CHLX adapted strains, the clinical strains of *K. pneumoniae* clinical ST258-KPC3 and ST846-OXA48, were exposed for two weeks to ¼ of their MIC of CHLX (9.8 and 19.5 μg/mL), respectively in liquid media with aeration. The antibiotic was replaced every 24 h. After these two weeks exposure, the two CA strains were obtained with MIC values of 39.1 and 78.2 μg/mL, respectively. 

Furthermore, to study the role of the plasmid (Figure S1) encoding the PemI/PemK TA system in the ST846-OXA48CA strain plasmids were removed from the strain. The cured plasmid ST846-OXA48CA CP strain of *K. pneumoniae* was generated following the protocol of El-Mansi et al. (2000) [44], where 3% sodium dodecyl sulfate (SDS) (10% *w*/*v* pH = 7.4) was used as a curing agent. To check the effective loss of the plasmid, PCR was carried out using the verification primers and, to corroborate the results, plasmid extractions were also performed and subsequently loaded on a 1% agarose gel.

All the bacterial strains and plasmids used in this study shown in the Table 3. 

### 5.2. Time-Killing Curve

The different time-killing curves were performed according to Hofsteenge et al. (2016) [8] in low-nutrient Luria-Bertani (LN-LB) broth (2 g/L tryptone, 1 g/L yeast extract and 5 g/L NaCl). The culture was incubated at 37 °C with shaking (180 rpm) until it reached the optical density at 600 nm (OD_600 nm_) of 0.6. At that moment, CHLX digluconate (10 × MIC) (Sigma-Aldrich, Darmstadt, Germany), IMP (50 × MIC) (Sigma-Aldrich, Darmstadt, Germany), or the combination of IMP (50 × MIC) and CHLX (10 × MIC), were added. Bacterial concentrations (CFU/mL) were determined at 0, 1, 2, 3, 4, 20, 24, 28, and 48 h by serial dilutions and plating on LB agar (10 g/L tryptone, 5 g/L yeast extract, 5 g/L NaCl, and 20 g/L agar). All experiments were performed in triplicates.

The MIC value for CHLX and IMP of the strains ST258-KPC3CA and ST846-OXA48CA are shown in Table 2 (MIC value of CHLX: 39.1 μg/mL and 78.2 μg/mL, respectively; and value of MIC of IMP of strain ST846-OXA48CA is 16 μg/mL). Therefore, 391 μg/mL and 782 μg/mL of CHLX was added in the time-killing curves in presence of CHLX digluconate (10 × MIC) in the strains ST258-KPC3CA and ST846-OXA48CA, respectively. In the case of the strains ST846-OXA48CA and ST846-OXA48CA CP, where the time-killing curves were performed in presence of (50 × MIC) IMP alone or in combination with (10 × MIC) CHLX, 800 μg/mL of IMP was added. 

### 5.3. Transcriptomic Study

The strain ST258-KPC3, ST258-KPC3CA, ST846-OXA48, and ST846-OXA48CA were cultured on solid LB plates, with or without CHLX according to the requirement of the strains and incubated at 37 °C for 24 h. One colony was removed and inoculated in liquid LB medium, with (1/16 × MIC) CHLX for the CA strain and incubated overnight at 37 °C with shaking (180 rpm). The inoculum was diluted (1:100) in LB media with (1/4 × MIC) CHLX for CA strains and without CHLX for wild-type strains. Then, when the culture reached a logarithmic growth phase (OD_600 nm_: 0.6), the RNA was extracted using the High Pure RNA Isolation kit (Roche, Mannheim, Germany), following the kit instructions, and the extract was treated with DNase (Roche, Mannheim, Germany). The RNA was subsequently quantified in a Nanodrop ND-100 spectrophometer (NanoDrop Technologies, Waltham, MA, USA) and was analyzed with an Agilent 2100 Bioanalyzer with RNA 6000 Nano reagents and RNA Nano Chips (Agilent Technologies, Santa Clara, CA, USA) to determine the quality and the integrity of the samples. All extractions were carried out for quadruple. After RNA extraction of the four replicates of each strain, rRNA and tRNA were removed using the Ribo-Zero rRNA removal kits (bacteria) (Illumina, San diego, CA, USA). The rRNA depletion was checked using Agilent RNA ScreenTape Assay (TapeStation 4200 Agilent, Santa Clara, CA, USA) and the RNA was quantified by Qubit TM RNA HS Assay Kit (Thermo Fisher Scientific, Waltham, MA, USA). Then, the transcriptomic libraries were performed using the Ion RNAseq Kit v2 in combination with the Ion Xpress TM RNA-Seq Barcode 01-16 Kit. Sequencing was carried out by emulsion PCR with Ion Sphere Particles (IPs). The enrichment of the library carrying IPs and the subsequent loading of the chip was developed in the Ion Chef automated system using the Ion PI Hi-Q Chef kit (Thermo Fisher Scientific, Waltham, MA, USA) and Ion PI chips v3 (Thermo Fisher Scientific, Waltham, MA, USA). Chip analysis was carried out on the Ion Proton sequencer (Thermo Fisher Scientific, Waltham, MA, USA). The generated data was analyzed using the specific software of the Torrent Suite 5.6.2 platform (Thermo Fisher Scientific, Waltham, MA, USA). The resulting readings (approx. 60 million) were exported in a FASTQ file using the FileExporter 4.6.0.0 (Thermo Fisher Scientific, Waltham, MA, USA) plugin. The alignment of the sequences against their respective controls was performed with the STAR software. Subsequently, the aligned readings were counted using the htseq-count software (HTSeq 0.6.1.p2). All data was normalized using DESeq2. 

### 5.4. Antimicrobial Susceptibility Test

MIC values of chlorhexidine, ciprofloxacin, tigecycline, tobramycin, imipenem, meropenem, gentamycin, piperacillin-tazobactam, ceftazidime, sulbactam, netilmicin, doxycycline, amikacin, minocycline, and colistin were determined by broth microdilution according to Clinical and Laboratory Standards Institute (CLSI) 2018 [46].

### 5.5. Construction of pCA24N (pemIK) and pCA24N (pemK)

The plasmids pCA24N (*pemIK*) and pCA24N (*pemK*) were constructed by amplifying the *pemI*/*pemK* module and the *pemK* gene in the expression vector pCA24N (cmR; LacIq) [45] inducible by IPTG (Fisher Scientific). The insertion of these genes was performed at the BseRI and NotI restriction sites under the control of T5-lac promotor (Table 4). Final constructions were verified by DNA sequencing. 

### 5.6. Toxicity Assay

The toxicity assay was performed as previously described Wood T.L. and Wood T.K. (2016) [47] with certain modifications. Overnight cultures of *K. pneumoniae* strains ST846-OXA48CA CP/pCA24N, ST846-OXA48CA CP/pCA24N (*pemIK*) and ST846-OXA48CA CP/pCA24N (*pemK*) were inoculated into 25 mL of LB broth medium with chloramphenicol (60 µg/mL; Sigma Aldrich, Darmstadt, Germany) to maintain the plasmid. IPTG (1 mM) was added when OD_600 nm_ was 0.3. For 200 min, the OD_600 nm_ was measured to determine growth evolution. All experiments were performed in triplicates.

### 5.7. Biofilm Formation Assay

The biofilm formation assay was performed in a 96-well polystyrene plate for 30 h. Briefly, cells of ST846-OXA48CA CP/pcA24N, ST846-OXA48CA CP/pCA24N (*pemIK*) and ST846-OXA48CA CP/pCA24N (*pemK*) were inoculated at OD_600 nm_ was 0.05 and incubated at 37 °C without shaking. After 6 h, IPTG at 1 mM was added and cells were incubated at 37 °C without shaking. 0.4 mM of IPTG was added every 8 h to avoid its degradation and to be able to observe the action of either the TA system or the toxin, as they are under an IPTG inducible promoter. Then, the cell density (OD_600 nm_) and total biofilm (OD_580 nm_) were measured by using 10% crystal violet staining, and quantified in a NanoQuant plate reader. Normalized biofilm was calculated by dividing the total biofilm by the bacterial growth for each strain. All experiments were performed in triplicates. 

### 5.8. Enzymatic Assay Using the Cell Proliferation Reagent WST-1

The cell proliferation/metabolic activity of the ST846-OXA48CA and ST846-OXA48CA CP strains in presence of the combination of IMP (50 × MIC) and CHLX (10 × MIC) was analyzed using a colorimetric enzymatic assay based on the water soluble tetrazolium salt (WST-1) reagent and electron mediators (Roche, Mannheim, Germany). Tetrazolium salts have become some of the most widely used tools in cell biology for measuring the metabolic activity of cells ranging from mammalian to microbial origin [48,49]. Briefly, the cultures of ST846-OXA48 and ST846-OXA48CA CP were incubated at 37 °C with shaking (180 rpm) until OD_600 nm_ was 0.6. At that moment the combination of IMP and CHLX, was added. After 24 and 48 h of antibiotic exposition and two washing, the culture cell (OD_600 nm_ = 0.1) was put in 96-well polystyrene plate (Corning Incorporated, NY, USA) and 10 μL of the reagent was added. After 1 h of incubation at 37 °C without shaking and 10 min with shaking (180 rpm), the optical density was measured at OD_480 nm_. The ST846-OXA48CA strain without antibiotic addition was taken as a control. The OD_480 nm_ of the medium culture (LN-LB) in the presence of WST-1 reagent was used to normalize all data. All experiments were performed in triplicates.

### 5.9. Statistical Analysis

Statistical analysis was based on the number of populations and comparisons. A Student’s *t*-test was used to compare two populations. All statistical analysis were performed using the GraphPad (Prism 8) software.

## Figures and Tables

**Table 1:** Gene expression in response to CHLX in the strain ST258-KPC3CA and ST846-OXA48CA.

Mechanism	Gene ^a^	Description	ST258-KPC3CA		ST846-OXA48CA	
			Log2 FoldChange	ID Gene	Log2 FoldChange	ID Gene
**Transporter**	smvA	Methyl viologen protein (cationic biocide resistance)	3.635	HGAILKPD_00917	1.209	EMNICGIE_00134
	actP	Acetate permease ActP (cation/acetate symporter)	2.724	HGAILKPD_00571	0.649	EMNICGIE_02128
	csbX	MFS superfamily	2.549	HGAILKPD_04325	0.618	EMNICGIE_04796
	lldP	L-lactate permease	2.486	HGAILKPD_04496	0.085	EMNICGIE_00243
	cysW	Ferric iron ABC transporter	2.181	HGAILKPD_02877	−0.266	EMNICGIE_02673
	potA	ABC transporter	1.749	HGAILKPD_02785	−0.351	EMNICGIE_03352
	pmrD	Signal transduction protein PmrD (colistin resistance)	-	-	2.360	EMNICGIE_04427
	pmrK	Polymyxin resistance protein PmrK (colistin resistance)	-	-	1.570	EMNICGIE_02839
**ATP metabolism**	atpD	ATP synthase beta chain	−0.209	HGAILKPD_02375	−0.232	EMNICGIE_00435
**TA systems**	ortT	Orphan toxin OrtT	0.731	HGAILKPD_02791	0.727	EMNICGIE_00095
	pemI	Programmed cell death antitoxin PemI	-	-	−0.100	EMNICGIE_05097
	pemK	Programmed cell death toxin PemK	-	-	−0.302	EMNICGIE_05098
**(p)ppGpp**	gppA	Guanosine-5′-triphosphate,3′-diphosphate pyrophosphatase	−0.765	HGAILKPD_02586	−0.399	EMNICGIE_03280
**ROS response**	cydA	Cytochrome d ubiquinol oxidase subunit I	1.318	HGAILKPD_03209	0.441	EMNICGIE_03423
	cybB	Cytochrome b561	0.456	HGAILKPD_02756	0.196	EMNICGIE_00060
**SOS system**	yedK	Putative SOS response-associated peptidase YedK	1.117	HGAILKPD_04848	0.003	EMNICGIE_04152
	yebG	DNA damage-inducible gene in SOS regulon	0.722	HGAILKPD_02193	0.455	EMNICGIE_04716

^a^ All gene expression showed have a *p*-value < 0.05, and overexpression and repression were considered from as a Log2fold change of at least 1.5 and 0.5, respectively. (-) Not detected. The rows shaded differently reveal those genes of high relevance in this study, the cationic biocide resistance gene, the colistin resistance genes, and the novel TA system PemI/PemK. Background colour indicates the genes of most interest from this study.

**Table 2:** MIC values (μg/mL) of different antibiotics for ST258-KPC3, ST258-KPC3CA, ST846-OXA48, and ST846-OXA48CA.

		MIC (μg/mL)													
Strain	CHLX	CIP	TGC	TOB	IMP	MRP	GEN	CAZ	TZP	SAM	NET	DOX	AMK	MIN	CST
**ST258-KPC3**	9.8	>32	2	64	4	8	4	>32	>32	1024	128	2	16	4	0.25
**ST258-KPC3CA**	39.1	>32	2	64	4	8	4	>32	>32	1024	128	2	16	4	0.25
**ST846-OXA48**	19.5	8	8	32	16	>32	32	>32	>32	128	16	8	2	8	0.5
**ST846-OXA48CA**	78.2	8	8	32	16	>32	32	>32	>32	128	16	8	2	8	16

CHLX, Chlorhexidine; CIP, Ciprofloxacin; TGC, Tigecycline; TOB, Tobramycin; IMP, Imipenem; MRP, Meropenem; GEN, Gentamycin; CAZ, Ceftazidime; TZP, Piperacillin-tazobactam; SAM, Sulbactam; NET, Netilmicin; AMK, Amikacin; MIN, Minociclin; CST, Colistin.

**Table 3:** Description of the bacterial strains and plasmids used in this study.

Strain or Plasmid	Main Characteristics	Source or Reference
ST258-KPC3	*K. pneumoniae* high-risk clone carrying β-lactamase KPC3	This study
ST258-KPC3 CA	*K. pneumoniae* high-risk clone carrying β-lactamase KPC3 adapted to CHLX	This study
ST846-OXA48	*K. pneumoniae* low-risk clone carrying β-lactamase OXA48	This study
ST846-OXA48 CA	*K. pneumoniae* low-risk clone carrying β-lactamase OXA48 adapted to CHLX	This study
ST846-OXA48 CA CP	*K. pneumoniae* low-risk clone carrying β-lactamase OXA48 adapted to CHLX and cured plasmid strain	This study
pCA24N	Expression plasmid Cm^R^, LacIq	[45]
pCA24N (*pemIK*)	Expression plasmid pCA24N with the TA systems *pemIK*	This study
pCA24N (*pemK*)	Expression plasmid pCA24N with the TA systems *pemIK*	This study

**Table 4:** Oligonucleotide used for cloning and sequencing.

Primer Name	Sequences	Sense	Reference
**Oligonucleotide for clonation**			
PemI_Fow(BseRI)	GAGGAGAAATTAACTATCATGCATACCACTCGACTG	5′-3′	This study
PemK_Fow(BseRI)	GAGGAGAAATTAACTATCATGGAAAGAGGGGAAATC	5′-3′	This study
PemK_Rev(NotI)	ATAAGAATGCGGCCGCCGCTCAGGTCAGGATGGTGGC	5′-3′	This study
**Oligonucleotide for sequencing**			
pCA24N Up	GCCCTTTCGTCTTCAC	5′-3′	This study
pCA24N Down	GAACTCCATCTGGATTTGTT	5′-3′	This study

## Supplementary Materials

The following are available online at https://www.mdpi.com/2072-6651/12/9/566/s1, Figure S1: Schema representative of the most relevant genes of the *K. pneumoniae* plasmid P2 (CP048940.1). This plasmid has more than 90% of homology by BLAST nucleotide (https://blast.ncbi.nlm.nih.gov/Blast.cgi) with the plasmid harbouring the pemI/pemK TA systems in the strain ST846-OXA48CA. This figure was created with the software Geneious version 2020.2 created by Biomatters. Available from https://www.geneious.com.
