# Supplementary material for: Mechanisms of Tolerance and Resistance to Chlorhexidine in Clinical Strains of Klebsiella pneumoniae Producers of Carbapenemase: Role of New Type II Toxin-Antitoxin System, PemIK
Source: Toxins (Basel). 2020 Sep 2;12(9):566. doi: 10.3390/toxins12090566 (PMC7551900; doi:10.3390/toxins12090566)
Supplement: Supplementary file 1 [file toxins-12-00566-s001.pdf]

# Supplementary Materials: Mechanisms of Tolerance and Resistance to Chlorhexidine in Clinical Strains of *Klebsiella pneumoniae* Producers of Carbapenemase: Role of New Type II Toxin-Antitoxin System, PemIK

**Ines Bleriot, Lucia Blasco, Mercedes Delgado-Valverde, Ana Gual de Torella, Anton Ambroa, Laura Fernandez-Garcia, Maria Lopez, Jesus Oteo-Iglesias, Thomas K. Wood, Alvaro Pascual, German Bou, Felipe Fernandez-Cuenca and Maria Tomas**

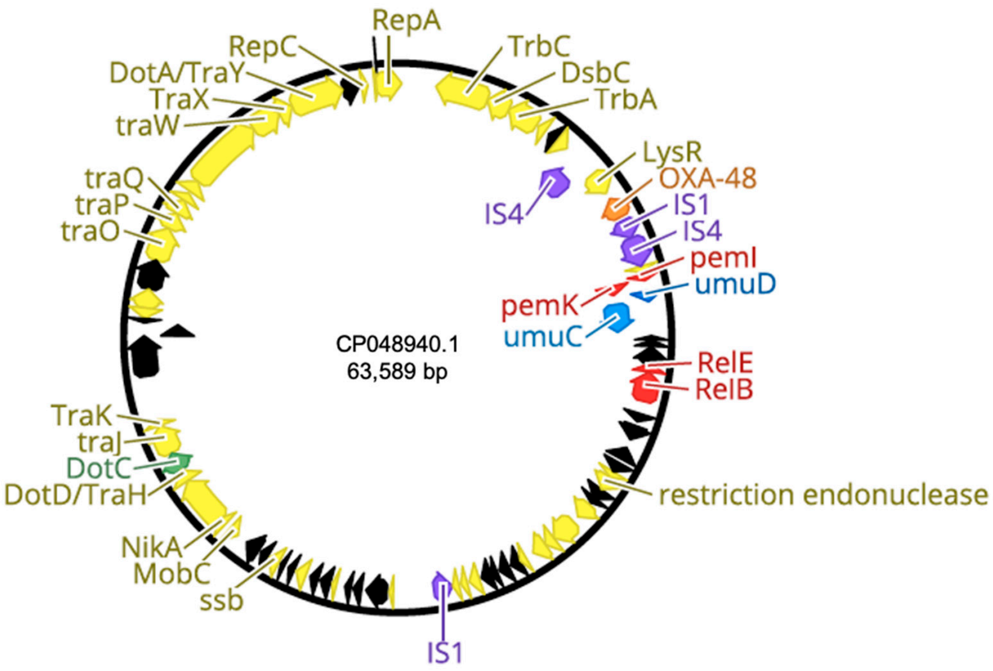


**Figure S1.** Schema representative of the most relevant genes of the *K. pneumoniae* plasmid P2 (CP048940.1). This plasmid has more than 90 % of homology by BLAST nucleotide (https://blast.ncbi.nlm.nih.gov/Blast.cgi) with the plasmid harbouring the *pemI/pemK* TA systems in the strain ST846-OXA48CA. This figure was created with the software Geneious version 2020.2 created by Biomatters. Available from https://www.geneious.com.
